# The role of latanoprost in an inflammatory bowel disease flare

**DOI:** 10.1093/gastro/gou044

**Published:** 2014-07-26

**Authors:** Sonali Paul, Martin Wand, Geoffrey T. Emerick, James M. Richter

**Affiliations:** ^1^Department of Gastroenterology and Hepatology, Tufts Medical Center, Boston, MA, USA, ^2^Department of Ophthalmology, University of Connecticut School of Medicine and Consulting Ophthalmologists, Farmington, CT, USA and ^3^Department of Gastroenterology, Massachusetts General Hospital, Boston, MA, USA

**Keywords:** lantanoprost, Crohn’s disease, prostaglandin, glaucoma

## Abstract

Ulcerative colitis and Crohn’s disease (CD) are characterized by inflammation of the intestinal mucosa and symptoms of abdominal pain and diarrhea. Many studies have shown an association between elevated levels of prostaglandins and mucosal damage in inflammatory bowel disease. We report a 50-year-old woman with a history of CD and open-angle glaucoma. Her glaucoma was treated with latanoprost, a prostaglandin analog, which was associated with an exacerbation of her CD. On discontinuation of latanoprost, her CD symptoms disappeared completely. This case suggests that in a patient with CD, topical administration of latanoprost may result in sufficient systemic absorption and circulation to promote a relapse of CD. This finding has important implications, not only for patients with inflammatory bowel disease and glaucoma, but also for both ophthalmologists and gastroenterologists.

## INTRODUCTION

Inflammatory bowel disease (IBD), including Crohn’s disease (CD) and ulcerative colitis (UC), are chronic inflammatory diseases of the bowel and clinically characterized by abdominal pain, diarrhea, and rectal urgency. The diseases are hypothesized to be related to dysregulatory immune responses to intestinal microbiota in a genetically susceptible host [1]. Many previous *in vitro* and *in vivo* studies have demonstrated elevated levels of mucosal and systemic prostaglandins (PG) in active IBD; [2–3] accordingly, patients are treated with medications that inhibit local prostaglandin synthesis.

We report a patient with a history of CD who developed an exacerbation of her disease with exposure to latanoprost, a PG F2-alpha analog commonly used in the treatment of glaucoma. Her symptoms improved upon discontinuation of the drug, further implicating the role of prostaglandins in CD. The patient gave informed consent for the publication of this report.

## CASE PRESENTATION

A 50-year old Caucasian woman presented with abdominal pain and hematochezia. A colonoscopy showed inflammation, confined to the sigmoid colon and rectum. She was diagnosed with CD and oral mesalamine was initiated. She achieved remission on this regimen for more than 11 years, never requiring systemic steroids. Twelve years after her CD diagnosis, she was diagnosed with open-angle glaucoma. She was treated initially with brinzolamide, a carbonic anhydrase inhibitor, but developed red eye and it was discontinued. Brimonidine tartrate (an alpha-2 adrenergic antagonist) was ineffective and she was started on latanoprost, initially in one eye, with good control of her intraocular pressures.

Within two weeks, however, she experienced abdominal pain and bloody diarrhea. A colonoscopy showed that her CD had progressed throughout her colon up to her splenic flexure. At the time she had no contacts with diarrheal illness and she denied a history of oversea travel. A work-up for infectious causes was negative, including cytomegalovirus and *clostridium difficile*. She had made no recent use of antibiotics and was on no new medications (including non-steroidal anti-inflammatory drugs or hormone replacement therapy). She began taking increasing doses of prednisone, reaching 60 mg daily within five months. Her symptoms improved but she required on-going oral steroids.

The patient’s glaucoma was under good control with lantanorprost and she began using it in both eyes. Within eight weeks of starting the drug in bilateral eyes, her CD began to flare again, with increasing abdominal pain and bloody diarrhea. She was kept on oral steroids, and rectal hydrocortisone and mesalamine were added. The regimen controlled her symptoms but she did not achieve remission. Further immunomodulator treatment with azathrioprine was being considered.

The patient and her ophthalmologist were aware of studies that showed enhanced production of PG during an active IBD flare, and were concerned that the intraocular exposure to a PG analog might be impacting her CD. Latanoprost treatment was discontinued and her symptoms disappeared. Her glaucoma was treated with istalol (a beta-adrenergic receptor blocking agent), achieving stabilization of her intraocular pressures. She has been symptom-free from her CD and kept on mesalamine and monthly certolizumab. She has not resumed latanoprost treatment.

## DISCUSSION

CD and UC are chronic inflammatory diseases of the colon and rectum. Several studies in both animal and human models have shown increased PG synthesis and concentrations in diseased tissue [2–3]. The treatment of IBD involves a variety of drugs that function to inhibit PGs [4]. In this case, a glaucoma patient with CD was treated with latanoprost, a topical PG, which aggravated her CD. Notably, withdrawal of this medication was associated with symptom improvement. It is certainly possible that the patient improved with the addition of certolizumab therapy (an anti-TNF agent) alone but this is difficult to prove at this point; however, in light of the known relationship between increased PG synthesis and bowel inflammation in CD, this clinical scenario points to an important adverse effect of a ‘benign’ glaucoma medication in patients with IBD.

Latanoprost, together with unoprostone, travoprost, and bimatorpost, represents the newest class of medications used in glaucoma management and all are derived from naturally occurring PG F2 [5]. They reduce intraocular pressure by increasing uveoscleral and aqueous outflow. Once applied, the drug is absorbed from the surface of the eye via the nasolacrimal duct into the inferior meatus of the nasal cavity [5]. The drug is rapidly broken down into the systemic circulation with a half-life of 17 minutes. Topical PGs can increase the concentration of endogenous PGs by stimulation of phospholipase A2 and release of arachidonic acid for PG synthesis [6]. Therefore, it is reasonable to hypothesize that administration of a PG F2 analogue might promote an inflammatory response in a susceptible person.

The low systemic concentrations and short half-life of topical PGs might account for low prevalence of systemic side-effects reported in the literature. There have been case reports describing exacerbation of angina with latanoprost use, which was reproducible with recurrent use [7]. In addition, there has been just one published case of a 39-year-old woman who developed abdominal cramps with topical travoprost [8]. Although a direct correlation cannot be established, the results of such case reports warrant further investigation on the pharmokinectics of this drug.

To obtain a measure of the possible relationship between ocular use of PGs and use of IBD medications, we reviewed a database of 20 million anonymous patient billing records, including medication prescriptions [9]. We found that, after a patient with known IBD was started on topical bimatoprost, use of IBD medications increased by 30% within three months [9].

This case, along with the others reported, shows a temporal association between use of a topical PG and exacerbation of IBD; however, such anecdotal reports do not prove a causal relationship. Since we know that a clinical trial of 50 000 patients is needed to detect an increase in adverse event incidence from 0.1 to 0.2% [10], and that systemic concentrations of topically applied PGs are low and transient, an infrequent adverse event might not be detected by current techniques. Nevertheless, a relapse into IBD is serious and raises important management considerations about patients who have both glaucoma and IBD, which apply to ophthalmologists, general practitioners, and gastroenterologists. Firstly, latanoprost and other such PGs, even in ocular form, should be prescribed with caution in patients with IBD. In addition, such medications should be covered by the list of questions asked when attempting to find the underlying cause of IBD exacerbations. This case demonstrates the importance of further study in this field.

**Funding:** Sonali Paul is supported by the 2013–2014 Bristol-Myers Squibb Virology Fellows Research Training Program. The manuscript received no direct financial support.

**Conflict of interest:** none declared.
